# ^129^Xe Nuclear Magnetic Resonance in Polymeric Membranes: A Computational Study of the Effect of Pore Size and Void Distribution on the Xenon Chemical Shift

**DOI:** 10.1021/acs.jpcb.5c05500

**Published:** 2025-09-19

**Authors:** Valerio Mazzilli, Carmen Rizzuto, Elena Tocci, Giacomo Saielli

**Affiliations:** 1 Institute on Membrane Technology, Unit of Padova − National Research Council of Italy (CNR-ITM), Via Marzolo, 1, Padova 35131, Italy; 2 Department of Chemical Sciences University of Padova, Via Marzolo, 1, Padova 35131, Italy; 3 Institute on Membrane Technology − National Research Council of Italy (CNR-ITM), Via P. Bucci 17C, Rende (CS) 87036, Italy

## Abstract

We present the results of a computational investigation of the structure and distribution of pore size in a polymer of intrinsic microporosity (PIM), a class of compounds with applications as sensors and membranes for gas separation. The high performance of PIMs, in our case PIM-EA-TB, meaning a PIM based on ethanoanthracene (EA) units linked by a Tröger’s base (TB, i.e., methanodiazocene), is largely due to the presence of interconnected micropores within the rigid polymer matrix. We have applied a computational NMR protocol based on a combination of MD simulations to generate several trajectories of xenon within the polymeric matrix and DFT calculations of the ^129^Xe chemical shift using clusters extracted from the MD trajectories. The comparison of experimental NMR data previously obtained and the results of the calculations allows to validate the bulk structure resulting from the MD simulations and to obtain a quantitative dependence of the ^129^Xe chemical shift on the distance of xenon from the pores’ internal walls. Such dependence is in very good agreement with the results reported in the literature concerning small model systems of Xe-alkane pairs and hints at a more general law that can be expected to hold for many different systems.

## Graphical abstract

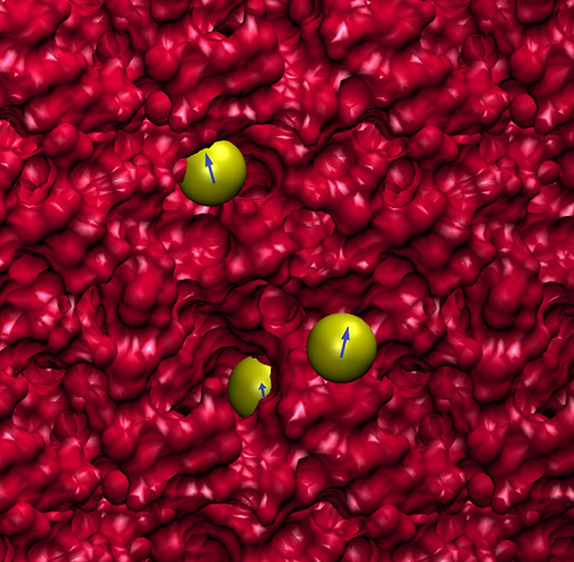

## 1 Introduction

^129^Xe NMR is a powerful technique for the investigation of the structural properties of various matrices using the noble gas as a probe.^1^ In fact, xenon is practically nonreactive and easy to detect from an NMR point of view since ^129^Xe has spin 1/2 and a relative abundance of 26.4%.^2^ More importantly, it is highly sensitive to the environment and it exhibits a relatively wide chemical shift range, up to 335 ppm in methylene iodide compared to free gas^3^ (excluding xenon covalent compounds^4^). Therefore, measuring the chemical shift δ­(^129^Xe) of xenon dissolved in a given system provides useful information on the chemical composition, structure, density, and porosity of the hosting matrix. Typical examples of the use of ^129^Xe NMR for structural studies of bulk phases and materials include molecular cages and cryptophanes,^5–7^ zeolites and other porous materials,^8,9^ polymers,^10^ simple liquids,^3,11–13^ liquid crystals,^14^ and ionic liquids.^15,16^

Although the experiment provides a substantial amount of structural information, interpreting the data is far from trivial. The measured chemical shift cannot be easily related to some structural arrangement since it is the result of a dynamic average over the distribution of positions explored by the probe nucleus within the hosting matrix, as well as the possible dynamics of the matrix itself in the case of fluid phases. It is therefore not straightforward to correlate a single number, δ­(^129^Xe), which potentially spans several hundreds of ppm, with the average structure or average distribution of Xe within the bulk phase.

In this scenario, computational NMR comes to the rescue. In fact, DFT (Density Functional Theory) calculations of the NMR properties (notably chemical shifts) of putative structures are now an established approach for validating or discarding structural hypotheses based on the level of correlation between the experimental and calculated data sets. Such protocols were pioneered about a quarter of a century ago by Bagno,^17^ Bifulco,^18^ Köck^19^ and Sebag,^20^ for organic molecules and natural substances and then further developed into sophisticated analytical tools by the groups of Goodman,^21,22^ and Sarotti.^23–25^ In these cases, the putative structure to be validated or retained based on the calculations is a covalent molecule, so the “space” of structural variation is discrete.

The same idea can be applied to the structural determination of complex fluids, for example, by comparing calculated chemical shifts along a Molecular Dynamics (MD) trajectory of suitable clusters extracted from the bulk at regular intervals. The final average value can be compared with the experimental results in order to assess the correct average bulk structure of the fluid as obtained from the given Force Field (FF) used in the MD simulation.^26^ Clearly, in such cases there is a continuum of possible “structures” depending on the set of FF parameters. At the same time, the reference molecule is very simple and fully characterized structurally. Such an approach has been successfully applied to pure ionic liquids,^27,28^ and their water mixtures,^29,30^ as well as to ^129^Xe dissolved in simple solvents^12^ and ionic liquids.^31^

Here, we aim to apply this computational protocol to the case of xenon dissolved in a polymeric membrane, in order to investigate the distribution of voids and porosity within the polymer. The purpose is to validate structural information, previously obtained from MD and experiments, by a direct comparison of calculated and experimental ^129^Xe chemical shifts.

Polymers of intrinsic microporosity (PIMs) represent a class of rigid, contorted polymers characterized by inefficient packing and large fractional free volume. Among them, PIM-EA-TB, composed of ethanoanthracene (EA) units linked by Tröger’s base (TB, i.e., methanodiazocene), has been widely investigated for its high gas permeability and microporosity stability.^32^ The structure of the repeating unit is reported in Figure 1.

**Figure 1:**
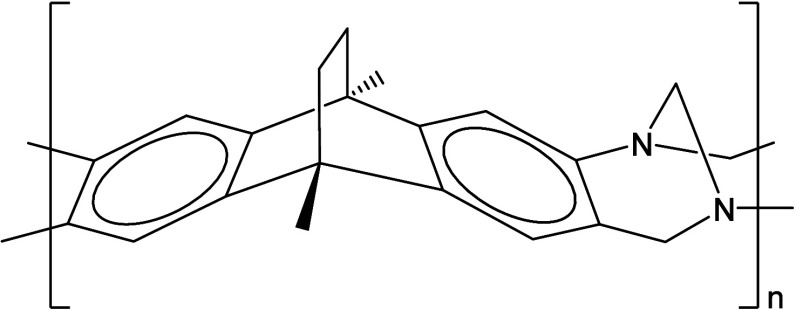
Chemical structure of the PIM-EA-TB repeating unit. Linear formula: (C_21_H_20_N_2_)_*n*_.

The combination of angular rigidity (TB unit) and bulky aromatic rings results in a highly microporous matrix with interconnected voids, which can be probed using xenon-based NMR techniques. In high-performance membranes, local void’s structure affects transport mechanisms at the molecular level. Understanding these effects through atomic-resolution probes such as ^129^Xe NMR can provide valuable insights into material design.

The study by Tocci et al.^33^ provided a comprehensive characterization of the void structure in a PIM-EA-TB polymeric membrane by combining geometrical void analysis based on the Hofmann–Heuchel method, Positron Annihilation Lifetime Spectroscopy (PALS), and experimental ^129^Xe NMR. In particular, ^129^Xe NMR spectroscopy revealed the presence of structurally diverse and accessible environments for xenon atoms, leading to a broad resonance with a line width of about 740 Hz. This diversity in local environments reflects the intrinsic heterogeneity of the pore architecture, as also suggested by the MD-derived cluster statistics. Overall, the integration of MD simulations with experimental techniques (PALS and ^129^Xe NMR) supports a consistent interpretation: the porous structure of PIM-EA-TB features a dual population of voids – small, isolated pockets and larger, partially connected domains.

However, on the one hand, both experimental techniques only provide indirect evidence of the structural features of the phase, while, on the other hand, MD simulations results, although offering an atomistic view, are limited by the accuracy of the used FF. It is therefore the aim of this work to provide a more direct link between the experimental results (in particular ^129^Xe chemical shift) and the structure obtained from the MD simulations by calculating the xenon NMR resonance through a combined computational protocol of MD simulation of the bulk phase structure and DFT calculations of the NMR shielding constant. The very good agreement obtained between calculated and experimental data will be used as a validation of the average bulk structure of the polymeric membrane, while, at the same time, it will offer a quantitative estimate of the dependence of the chemical shift on the size of the voids hosting the probe atom.

## 2 Computational Details

### 2.1 MD Simulations: Polymeric Model Construction

The MD simulations were performed using the Materials Studio package from BIOVIA^34^ and employed the COMPASS FF.^35,36^ A polymeric chain consisting of 15 monomer units served as a template for the initial packing, with the Amorphous Cell module of the BIOVIA software. This process involved constructing chains within a cubic periodic cell at 298 K, utilizing a RIS-based bond-by-bond construction method while considering bond torsion probabilities and bulk packing requirements.^33,37,38^ The Theodorou/Suter method,^39,40^ implemented in the Amorphous Cell module, was used to build the amorphous packing by inserting one segment per Monte Carlo step. The conformations were sampled based on Flory’s Rotational Isomeric State (RIS) theory.^41^ In each packing model, five polymeric chains grew together at an initial reduced density (relative to the experimental value) of 0.1 g/cm^3^. Each simulation cell also contained 400 randomly distributed argon atoms as spacer molecules. These spacers were included to avoid artifacts such as catenated rings or the entanglement of side groups and backbone chains through ring substructures, following a procedure well-established in the literature.^42^ The spacers were then removed during the equilibration of the polymer models. The simulation boxes were then added with 1, 3, and 10 Xe atoms, which represented the different loadings studied. Each simulation box was then equilibrated using a multistep molecular dynamics protocol at 303 K and 1 atm, with a time step of 1 fs and employing the Berendsen thermostat and barostat (time constant = 0.1 ps).^43^ A cutoff distance of 1.25 nm with a spline width of 1 Å was used to treat the van der Waals and the electrostatic interactions using a group-based summation method.^44^ The final systems achieved a density of 1.02 g/cm^3^ and contained approximately 3266 to 3275 atoms, depending on the xenon content. To enhance statistical accuracy, five independent boxes were created for each xenon loadings (1, 3, and 10 atoms), totaling 15 boxes. Production MD trajectories were generated over 4 ns at 303 K and 1 atm pressure, using a time step of 1 fs, with configurations saved every 200 ps for further analysis of the different boxes and xenon concentrations.

To reference the xenon chemical shift, additional simulations were performed on a system containing one xenon atom in a box of 250 hexane molecules. This system was simulated using GROMACS software^45^ in the NpT ensemble at a temperature of 300 K and pressure of 1 bar for 60 ns, with the last 30 ns for production. The time step was set to 1 fs, the cutoff was 1 nm, and the Berendsen barostat and thermostat were used. The FF parameters were derived from previous research.^46,47^

As an example, a simulation box of Xe@PIM with the highest Xe content is shown in Figure 2.

**Figure 2:**
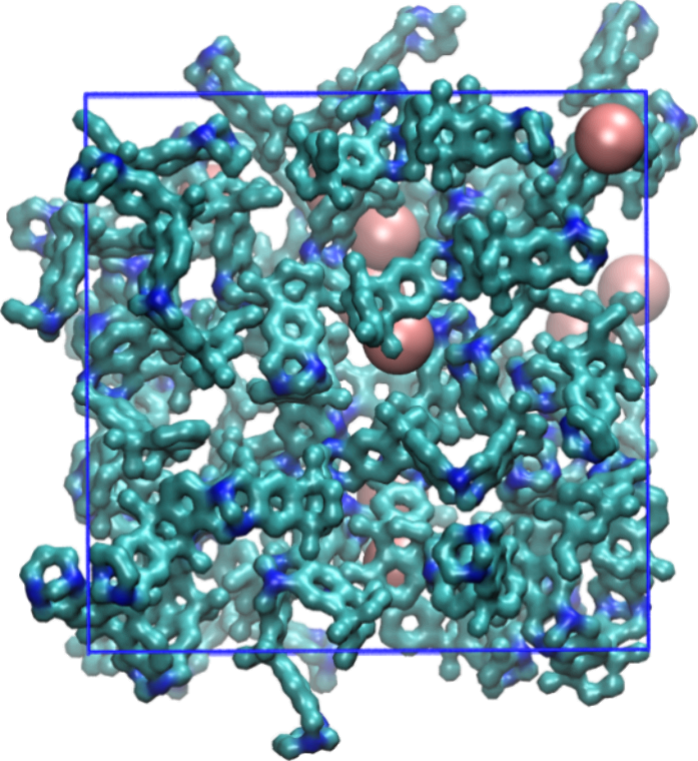
Simulation box of the system with 10 xenon atoms in the PIM-EA-TB matrix. C and N are represented in cyan and blue, respectively. Xe atoms are pink spheres, hydrogens are omitted for clarity. Voids ca. be clearly seen in the polymeric structure.

### 2.2 Radial Distribution Function

The radial distribution function, *g*_*AB*_(*r*), between particles of A and B is defined as follows (eq 1):

$$g_{AB}\left(r\right)=\frac{\left\langle g_{B}\left(r\right)\right\rangle }{\langle g_{B}\rangle_{local}}=\frac{1}{\langle g_{B}\rangle_{local}}\frac{1}{N_{A}}\overset{N_{A}}{\underset{i\in A}{\sum }}\overset{N_{B}}{\underset{j\in B}{\sum }}\frac{\delta \left(r_{ij}-r\right)}{4\pi r^{2}}$$ 1

where ⟨*g*_*B*_(*r*)⟩ is the particle density of type B at a distance *r* around particles A, and ⟨*g*_*B*_⟩*_local_* is the particle density of type B averaged over all spheres around particles A having radius rmax. The radial distribution function between Xe and the non-hydrogen atoms of the polymer (C and N) is employed to evaluate the local environment. The position of the first minimum in the RDF (at ∼ 7 Å) was used as the cutoff radius for cluster definition to be used in the DFT calculations.

### 2.3 Cluster Extraction for ^129^Xe Chemical Shift Calculations

The chemical environment of Xe atoms was assessed via clusters extracted from the MD trajectories. Only monomer fragments containing atoms within the cutoff were retained in the clusters: this means that if an atom, different from hydrogen, has a distance from xenon shorter than the cutoff radius, the residue to which that atom belongs to, is fully included into the cluster. Since the polymer is made of repeating monomeric units, only the closest units are used for the DFT calculations. Bonds connecting a selected unit to neighboring monomers were replaced with hydrogen atoms to preserve chemical valency and avoid artifacts in DFT calculations. This approach ensured consistent chemical topology across all clusters. In Figure 3 the structure of the monomer is depicted with the hydrogens used as substitutes for the C–C bonds between monomers explicitly indicated.

**Figure 3:**
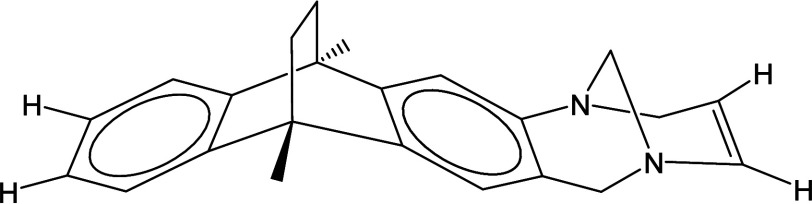
Structure of a monomer of PIM-EA-TB in which the bonds of the carbons with the previous and subsequent units are replaced with bonds with hydrogens, explicitly indicated. These positions represent the cutting points in the polymer chain to make the cluster surrounding the Xe atom for the DFT calculations.

Cluster extraction was performed every 200 ps for each Xe atom in the simulation, resulting in 100 configurations for the system with one Xe atom (20 configurations in each box times 5 independent boxes), 300 configurations for the system with three Xe atom and 1000 configurations for the systems with ten Xe atoms dissolved. The average size of the clusters was 325 atoms, with the smallest being 132 atoms and the largest 611 atoms (including hydrogens).

### 2.4 DFT Calculations of ^129^Xe NMR Shielding Constants

DFT calculations of ^129^Xe shielding constants were performed using the Amsterdam Density Functional (ADF 2017) software.^48,49^ All calculations included scalar relativistic effects via the Zeroth Order Regular Approximation (ZORA).^50^

Following our previous works,^4,31^ we adopted the BLYP functional, a generalized gradient approximation (GGA) combining Becke’s exchange^51^ and the Lee–Yang–Parr correlation terms,^52,53^ as a reliable compromise between computational efficiency and accuracy in predicting ^129^Xe NMR shielding constants. It is now well established that hybrid functionals generally offer superior accuracy in the prediction of NMR properties compared to GGA functionals, although at significantly higher computational cost. In particular, the BHandHLYP hybrid functional has been found to produce ^129^Xe chemical shifts that closely match high-level CCSD­(T) results for model systems such as the Xe–benzene dimer.^54^

To assess the potential benefits for our systems, in a previous work we benchmarked BHandHLYP against BLYP on a selected set of clusters derived from Xe@dihalomethane simulations.^12^ The hybrid functional yielded chemical shift values in excellent agreement with experimental data, however, the computational demands were substantial: each BHandHLYP calculation required between 20 and 100 times more CPU-time than a corresponding BLYP calculation, for clusters containing several hundred atoms, as it is the case here.^12^ Given the large number of clusters involved in our study (1400 in total), we opted for the BLYP/QZ4P­(Xe) level of theory as the best compromise between computational efficiency and accuracy. Accordingly, ^129^Xe atoms were treated with the QZ4P basis set, while surrounding carbon and nitrogen atoms were modeled using frozen-core TZP.1s, and hydrogen atoms with TZP. This combination ensured consistent treatment of the local environment while keeping computational cost manageable.

The chemical shift of xenon in our PIM-EA-TB system, δ_*Xe* @ *PIM*_, is defined as the difference between the shielding constant of Xe in gas phase σ_*Xe*(*g*)_ and the shielding constant of Xe dispersed within the polymer, as generated by MD simulations, σ_*Xe* @ *PIM*_, according to eq 2:

$$\delta_{Xe@PIM}=\frac{\sigma_{Xe\left(g\right)}-\sigma_{Xe@PIM}}{1-\sigma_{Xe\left(g\right)}}\approx \sigma_{Xe\left(g\right)}-\sigma_{Xe@PIM}$$ 2

where the denominator can be approximated to 1 since shielding constant of Xe are of the order of 10^–3^. The calculation of the shielding constant of gaseous xenon, taken as the reference, should be obtained, in principle, from the calculation of Xe in vacuum, that is a purely single-point DFT calculation of an isolated atom. To minimize systematic errors, we also employed a relative shift scheme using Xe@hexane as a secondary reference system. The experimental chemical shifts can then be compared by simply calculating the differences of the experimental chemical shifts of the Xe in the two different media.

### 2.5 Void Space Analysis

To evaluate the porous structure of the polymer matrix, void space analysis was carried out on the equilibrated MD boxes using Zeo++.^55^ For these analyses the xenon atoms were removed from the boxes. This open-source software applies a Voronoi tessellation algorithm to calculate key geometric descriptors of the free volume. Three pore metrics that were extracted for each configuration are (see Figure 4): *D*_*i*_: Diameter of the largest included sphere within a cavity; *D*_*f*:_ Diameter of the largest free sphere that can pass through the network; *D*_*if*_: Diameter of the largest sphere that can move along a continuous path.

**Figure 4:**
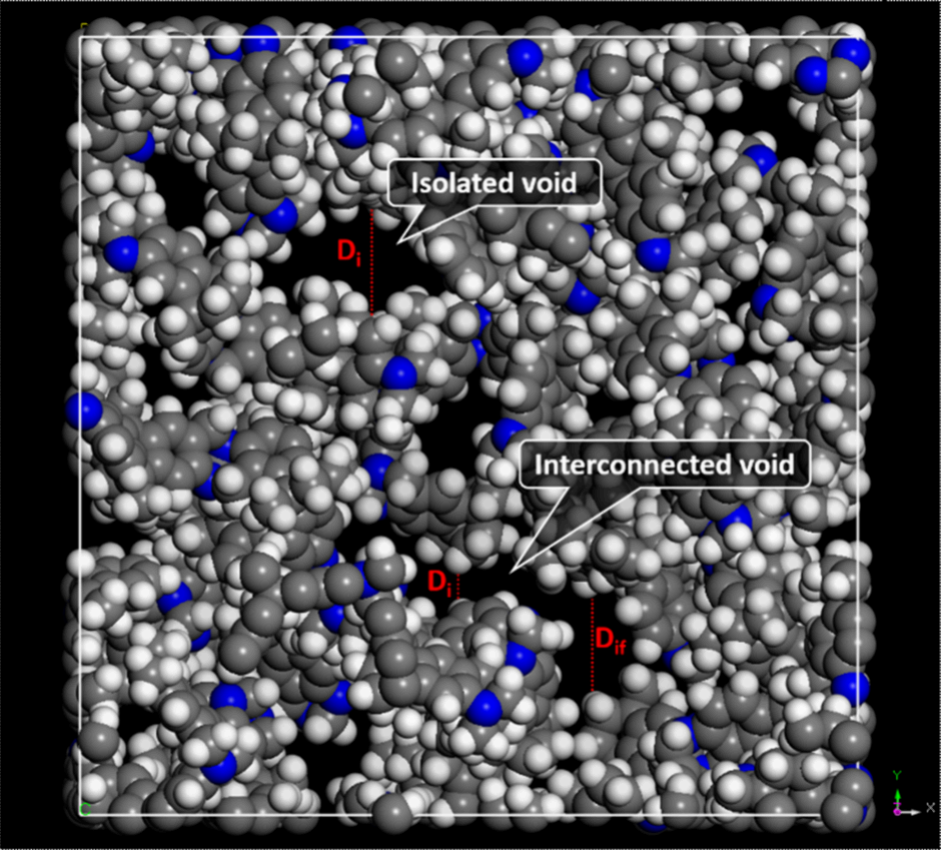
Example of different pore diameters as obtained using Zeo++ software. Isolated and interconnected voids are highlighted.

A point-like probe of radius of zero Å was used, and the analysis was conducted on the five MD-equilibrated boxes for each loading. Average and standard deviation values were reported for each metric and correlated with xenon distribution and NMR chemical shift data.

## 3 Results

### 3.1 Morphology and Microporous Structure of the PIM-EA-TB Matrix

The morphology of the polymer was assessed by analyzing the simulation boxes and the voids distribution. A qualitative visualization of the free volume elements is shown in Figure 5, composed of thin slices (∼3.7 Å) perpendicular to the *z*-axis.

**Figure 5:**
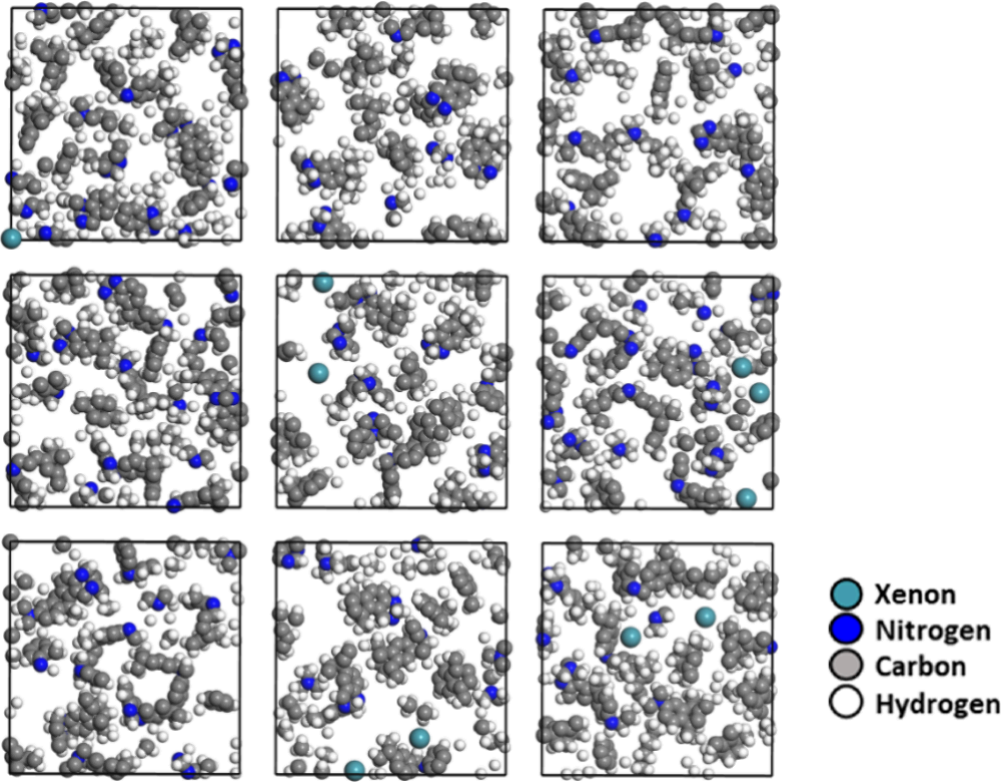
Representative slices, centered at different levels along the *z* axis of the box, of one packing model with thickness 3.7 Ǻ.

The irregular and nonspherical shapes of the empty regions are consistent with the high free volume characteristic of PIMs. Some of these cavities span multiple slices, indicating significant connectivity and extended pore domains reflecting a structurally complex and heterogeneous porosity. This behavior is typical of polymers with intrinsic microporosity and is consistent with previous findings using the Hofmann-Heuchel approach.^33,56,57^

Geometric analysis using Zeo++ simulations provided values for the maximum cavity diameter *D*_*i*_ ≈ 7.91 Å, the largest sphere that can move continuously through the porous network, representing the narrowest bottleneck *D*_*f*_ ≈ 2.73 Å, and the largest sphere that can be included along the percolating path *D*_*if*_ ≈ 7.20 (see Table 1).

**Table 1:** Average Dimensions of the Pore Diameters

average pore metrics	average (Å)	std. dev. (Å)
*D*_*f*_	2.68	0.23
*D*_*i*_	7.64	0.98
*D*_*if*_	7.20	1.16

These values, although derived from localized structures, align well with those obtained by Tocci et al.,^33^ reinforcing the description of a hybrid porosity: composed of relatively large cavities, yet with limited long-range connectivity. However, it should be noted that these results are based on a fixed spherical probe and a static Voronoi tessellation, which may not fully capture the true accessibility or dynamic nature of the porous network, especially at the interfaces between connected and isolated void domains. The study by Tocci et al.^33^ provided an *R*_max_ distribution, i.e., the smallest sphere that can be placed within the voids, at around 2.6 Å, consistent with the PALS R3 parameter (≈2.52 Å).

Figure 6 indicates the global distribution of pore sizes throughout the polymer matrix, as computed with Zeo++. The *y*-axis has been normalized to the frequency counts of each pore diameter, meaning that the histogram reflects how often each pore size occurs across the sampled volume. However, this representation does not resolve individual cavities or assign pore sizes to specific connected regions.

**Figure 6:**
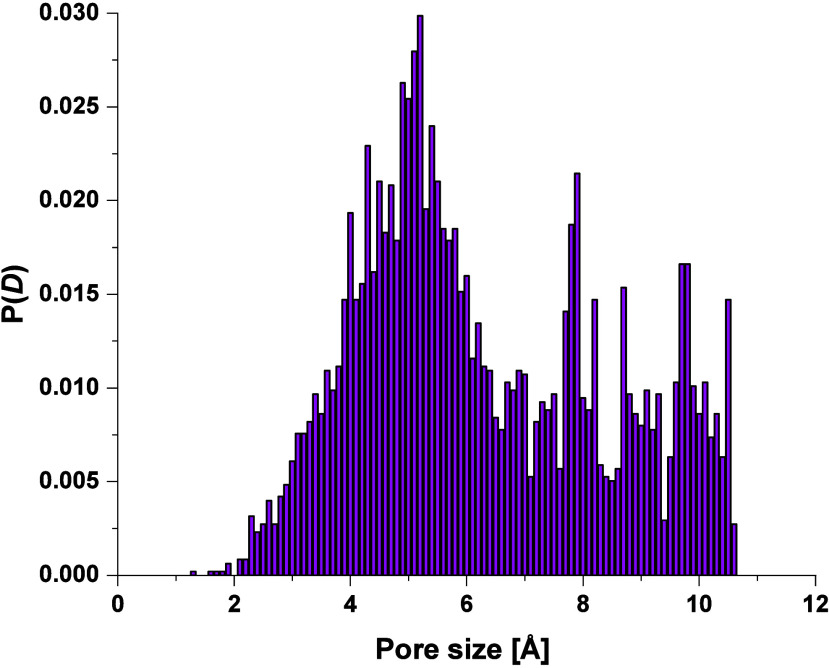
Pore size distribution of the PIM-EA-TB investigated. All pores’ dimensions (*D*_*f*_, *D* *i*, and *D*_*if*_, see Table 1) are considered.

This contrasts with more refined descriptors such as the Heuchel–Hofmann Rmax and Vconnect metrics, which explicitly segment the porous domain into distinguishable accessible clusters and characterize their size and connectivity. In particular, the Vconnect distribution, quantifying the maximum spatial extent of each connected void region, exhibited a broad profile with a main peak around 15 Å and shoulders near 10 Å, highlighting the presence of extended but topologically diverse pore domains.^33^ This reflects the structural heterogeneity of the polymer matrix, suggesting that, while many small voids exist, a non-negligible fraction of the porosity arises from larger, interconnected free volume regions, potentially relevant for guest diffusion and transport.

### 3.2 Xenon Environments and Chemical Shift Distributions

As shown in Figure 7a), the local environments around Xe atoms exhibit a characteristic first minimum in the RDF at ∼ 7 Å, confirming the spatial extent of the nearest neighbor shell. The extracted clusters, exemplified in Figure 7b), reveal a diverse set of atomic configurations around Xe, reflecting the inherent structural heterogeneity of the polymeric matrix.

**Figure 7:**
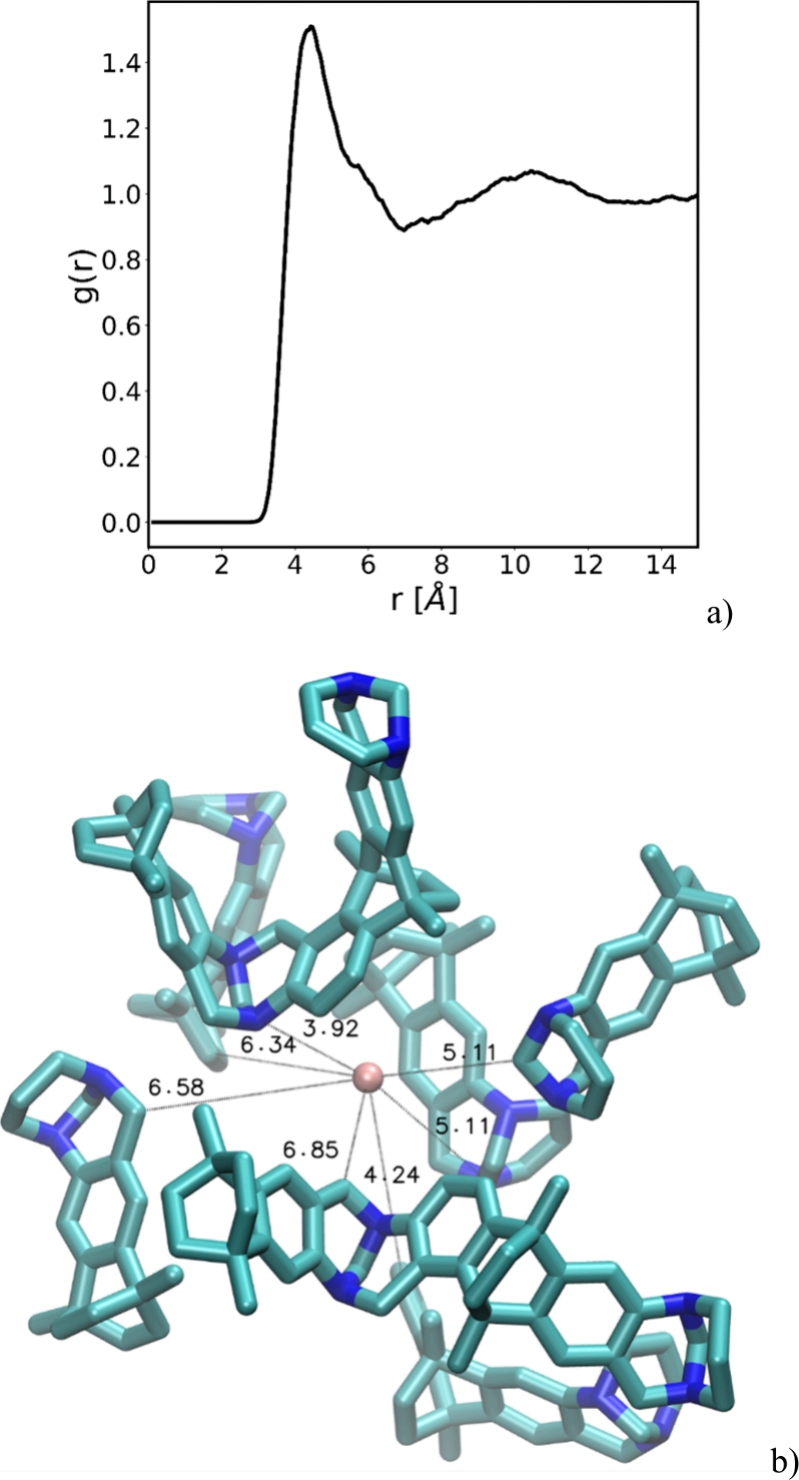
(a) Radial distribution function calculated between the Xe and all the C and N present in the systems. (b) Sample of an extracted cluster comprising Xe, in the center, surrounded by monomers and oligomers with the distances of the closest residues indicated in Å.

The intrinsic microporosity of the polymer and its void distribution allow Xe atoms to experience the cavities, pockets and empty regions of the polymeric matrix. Different environments affect the ^129^Xe NMR properties influencing the calculated ^129^Xe chemical shifts, as proximity to surrounding atoms enhances deshielding effects, leading to higher shift values. In fact, it is well-known in the literature that the deshielding effect is mostly the result of noncovalent interactions, in particular the “contact” repulsion between the electronic clouds, due to the Pauli exclusion principle (the so-called steric interaction), between the xenon atom and the solvent molecule.^3,58,59^

Specifically, clusters in which atoms are positioned closer to the Xe nucleus exhibit pronounced deshielding effects, resulting in larger chemical shift values. The distribution of the calculated chemical shifts is reported in Figure 8 where the system with different Xe concentrations is evaluated both separately, for each loading, and as a whole. The distribution of shifts appears unsymmetrical with a peak at low chemical shifts and a long tail at higher chemical shifts. The mean values, reported in the legend of Figure 8, are obtained as the average of all the calculated chemical shifts of the systems having the same Xe content. Since the produced configurations follow the proper partition function of the ensemble in which they are simulated, the sampling is considered uniform and for this reason the chemical shift is calculated as an arithmetic mean.

**Figure 8:**
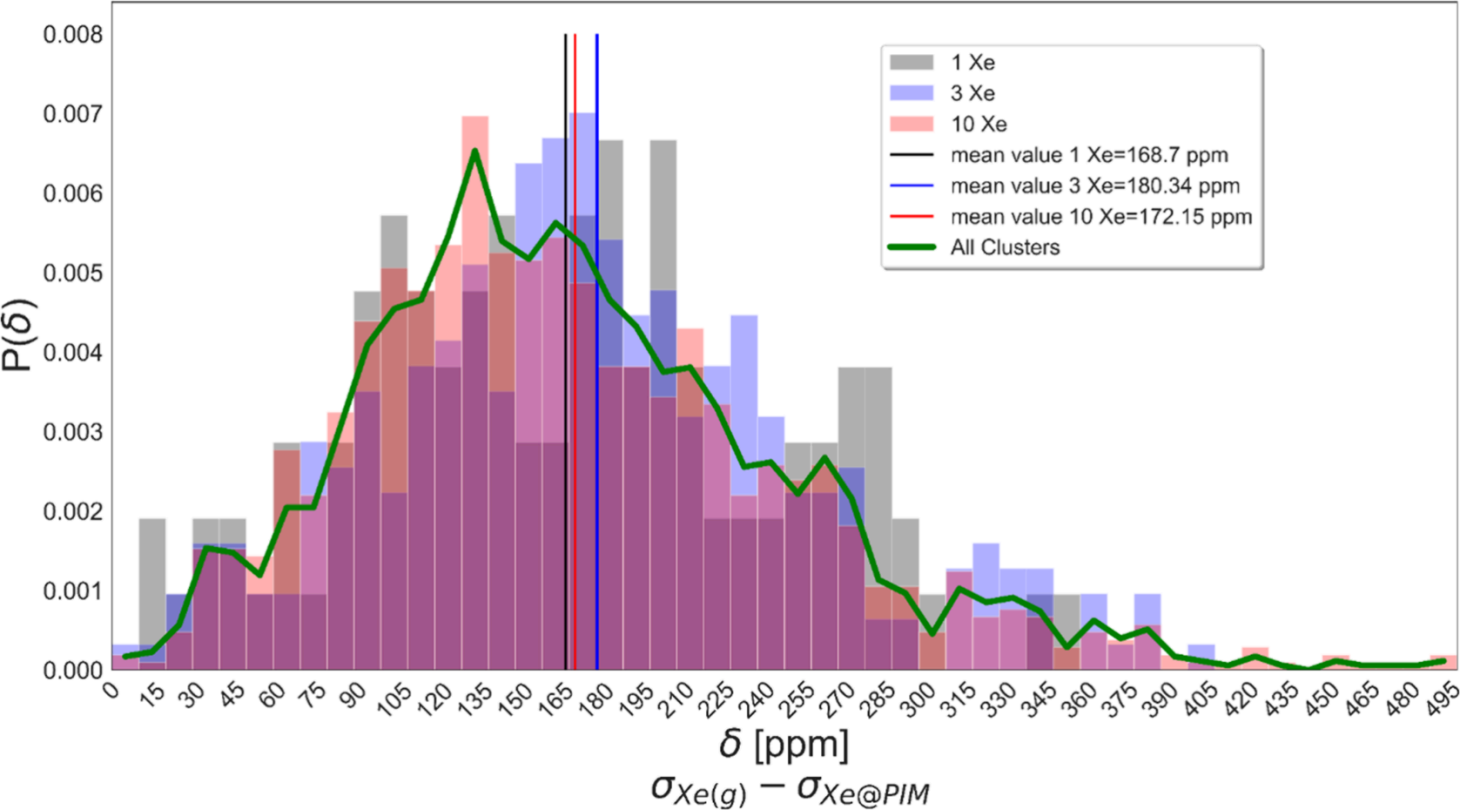
Probability distribution of the calculated chemical shifts of the systems with 1, 3, and 10 Xe atoms depicted with gray, blue and red bars respectively. The green curve is the probability distribution of all the calculated chemical shifts. Mean values for 1, 3, and 10 Xe atoms are the black, blue and red vertical lines with the value indicated in the legend. Superposition of bars produces different colors.

If the chemical shifts are calculated as the difference between the shielding constant of Xe in vacuum and the shielding constant of Xe in PIM (σ_Xe(g)_-σ_Xe@PIM_), the obtained chemical shift values for the systems with 1, 3, and 10 Xe are 168.7, 180.34, and 172.15 ppm, respectively. First, we note that there is not a clear trend with the Xe loading, at least for these three xenon concentrations. In fact, the three values are quite close and suggest that there is no dependence of the average chemical shift on the loading, in our simulations. The values, however, as well as the average of the three, are higher than what is found in the literature experimentally, that is 109.7 ppm,^33^ with a discrepancy of more than 70 ppm on the calculated values. Interestingly, setting as a reference for the chemical shift the shielding constant of Xe in a different system, evaluated with a similar computational MD+DFT protocol, significantly improves the results. If we employ as reference the shielding constant of Xe in hexane (σ_Xe@hexane_), the chemical shift obtained has an error of just few ppm, depending on the slightly different experimental values used. The shielding constants of Xe@hexane are obtained using the same level of theory employed for the calculation of the Xe@PIM shielding constant and the difference is compared to the difference of experimental chemical shifts. All these values are reported in Table 2. The different shielding reference clearly cancels out, in the final chemical shift, some systematic errors that are introduced in the evaluation of the shielding through a complex MD+DFT protocol and that are absent in the calculation of the shielding of free xenon gas.

**Table 2:** Chemical Shifts of Xe@PIM Calculated as σ_Xe‑gas_–σ_Xe@PIM_ and as σ_Xe@PIM_–σ_Xe@hexane_ ^d^

# Xe	δ_calc_ [ppm]^a^ σ_Xe(g)_-σ_Xe@PIM_	δ_calc_ [ppm]^b^ σ_Xe@hexane_-σ_Xe@PIM_
1 Xe	168.7	–50.58
3 Xe	180.34	–38.94
10 Xe	172.15	–47.13
mean value^c^	173.66	–45.62
δ_exp_(Xe@PIM)	109.7 ^33^	–41.5 ^60^ /–51.8 ^11^

a With respect to free xenon gas.

b With respect to Xe@hexane.

c Weighted average considering weights as 1/(10 + 3+1), 3/(10 + 3+1), and 10/(10 + 3+1) for 1, 3, and 10 Xe atoms, respectively. Experimental data from ref 60 (151.2 ppm w.r.t. free gas) is measured at 295 K. Experimental data from ref 11 (161.5 ppm w.r.t. a reference frequency of the isolated xenon ν_0_ = 24,774,801 Hz) is measured at 298 K.

d The experimental chemical shift to be used for comparison and referenced to free gas and hexane is reported in the last entry.

This level of theory, which makes use of a Xe@alkane reference, provides very satisfactory results for similar noncovalently interacting xenon environments, such as those encountered in polymeric cavities and liquid hexane, with an affordable computational cost.

### 3.3 Chemical Shift vs Local Geometry

The main contribution to the shielding values, and thus to the chemical shift, is ascribed to the close surroundings of Xe atoms. The calculated data, reported in Figure 9, although being quite scattered can be used to estimate the dependence of the chemical shift on the size of the voids (taking the average distance of the four closest non-hydrogen atoms to reduce the scattering of the data).

**Figure 9:**
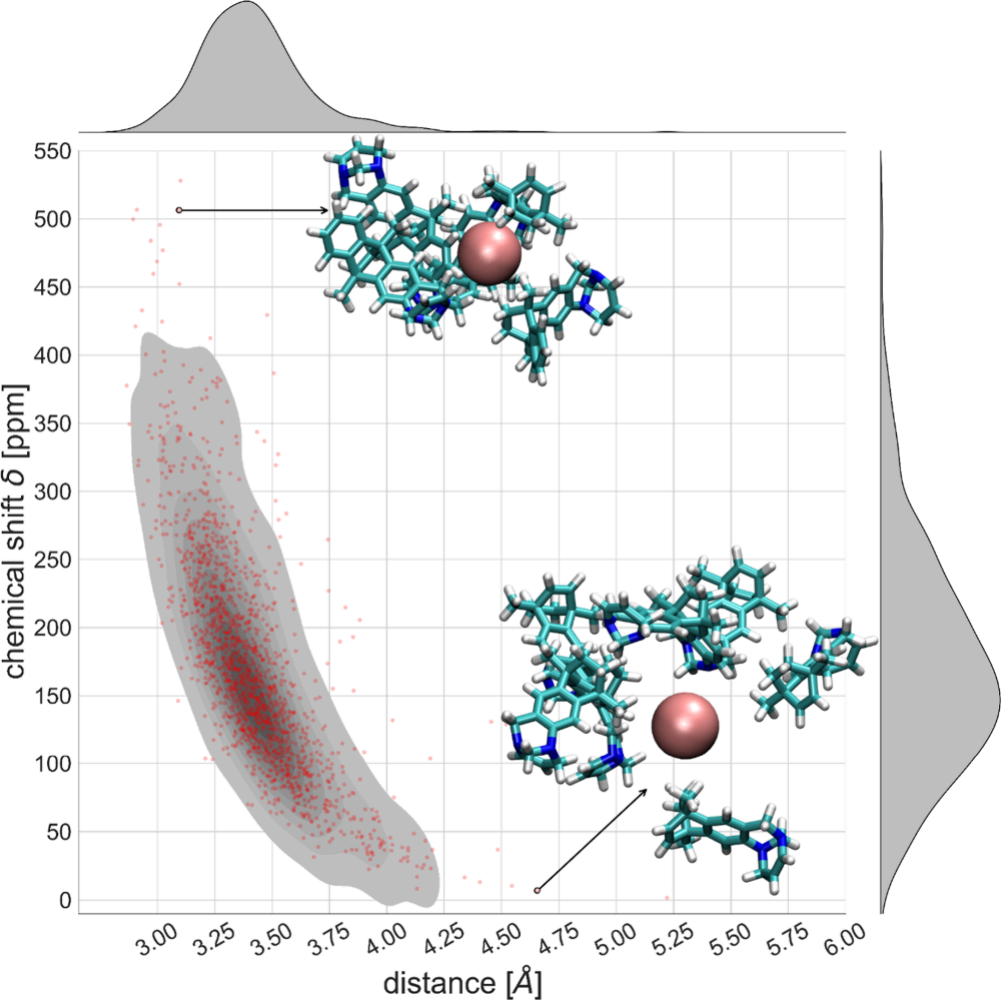
Dependence of the chemical shifts as a function of the mean distance of the four closest atoms to the central Xe. Grayscale indicates the more populated area of the plot. On the right and top axis, chemical shift and distance distributions with respect to the central Xe atom, respectively, are shown. Two examples of clusters are reported for the high shift region, top, and low shift region, bottom.

It can be seen that the clusters having a large chemical shift value are the ones with atoms closer to the Xe, while shift values close to 0 ppm are found for Xe being in an almost empty region of space. In Figure 9 two example clusters are reported for the high shift and low shift case, indicating that for low shift values the Xe does not have any atom within ∼ 4.5 Å around it.

When close to other nuclei, the Xe is more deshielded, producing higher chemical shift values like in the case of the cluster represented at the top of Figure 9.

The effect of Xe content on the shifts is found not to strongly depend on Xe concentration, but this holds true also for experimental evidence, where the chemical shift of ^129^Xe increases only by about 10 ppm with the increasing of Xe nominal pressure from 0 to 110 kPa.^33^

Finally, the observed relationship between δ­(^129^Xe) and the mean distance to the four closest polymer atoms seems to follow an exponential decay, δ = *A*·exp­(−*r*/*r*_0_). In Figure 10 we report the same data as in Figure 9, but in log scale. The results are linearly fitted testing two types of weight for the residues, one with no weight and a second one with a weight inversely proportional to the calculated values. The decay constant, *r*_0_, which is simply the reciprocal of the slope, is 0.52 Å and 0.42 Å for no weight and reciprocal weight, respectively. The corresponding amplitudes *A*, obtained from the linear fitting, are 1.05·10^5^ ppm and 4.87·10^5^ ppm, respectively.

**Figure 10:**
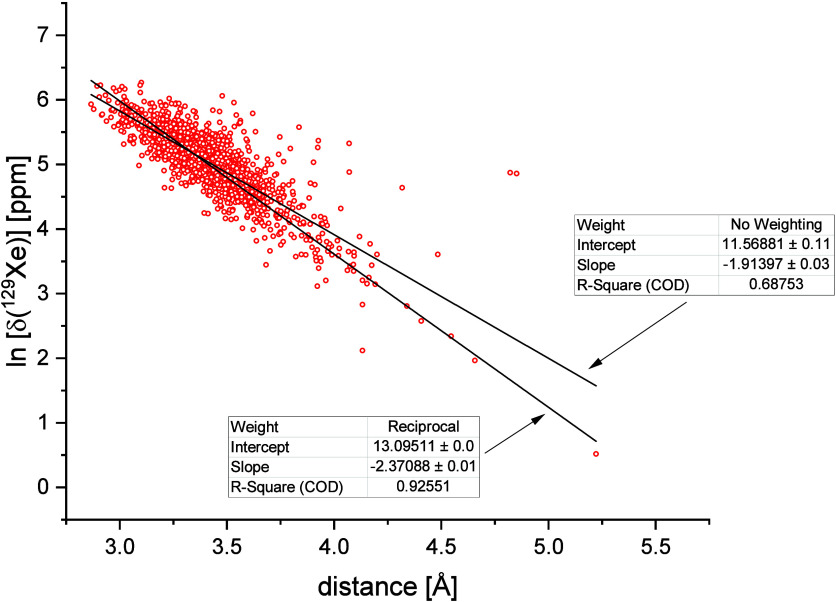
(Red circles): calculated chemical shifts of ^129^Xe in the PIM-EA-TB matrix as a function of the distance from the pore’s wall (average of the closest four atoms). (Solid back lines) linear fitting with two different weightings of the residues. Fitting parameters are in the insets.

This result, taking into account the large scattering of the points, is in excellent agreement with previous data concerning the decay of xenon chemical shift with the distance in model pairs of a Xe atom and short alkanes arranged as in Figure 11.^61^

**Figure 11:**
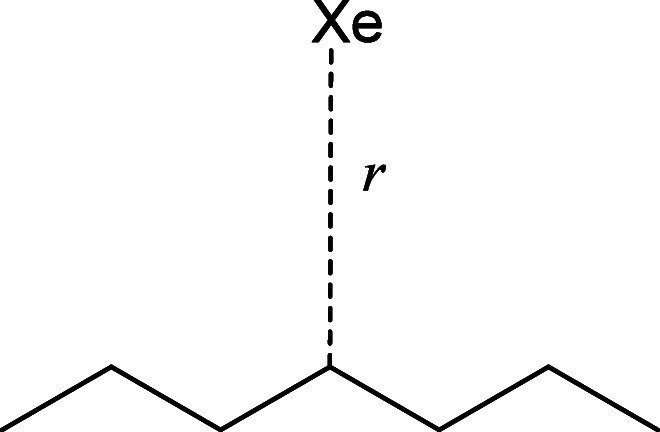
Model Xe···alkane pair investigated in ref 61. The chemical shift of Xe was found to exhibit an exponential decay δ = *A*exp­(−*r*/*r*_0_) with a decay constant *r*_0_ of 0.47 Å and an amplitude depending on the number methylene groups interacting with Xe through van der Waals interactions.

The decay constant of 0.47 Å found in ref.,^61^ is practically the same as found here, while the amplitudes were found in ref.^61^ to follow a roughly additive rule with the number of CH_2_ groups facing xenon. In fact, amplitudes (all in 10^5^ ppm) were 1.97, 1.93, 1.81, 2.38, 3.02, 2.96 and 2.92, from methane to heptane, respectively,^61^ also in agreement with the results obtained for ^129^Xe trapped in the voids of PIM-EA-TB. Such perfect correspondence highlights a general and universal trend of the chemical shift of xenon as a function of the distance from alkylic groups. Unfortunately, the chemical shift of ^129^Xe in the real voids of the PIM-EA-TB matrix cannot be expected to strictly follow the same dependence as the small model system, due to additional effects related with the complex geometries and structure of the walls of the voids, as well as the presence of nitrogen atoms (not considered in the simple model systems of ref.^61^

## Conclusions

In this study, we applied a combined computational protocol integrating molecular dynamics simulations and relativistic DFT calculations to explore the ^129^Xe NMR response in a PIM-EA-TB membrane matrix. This multiscale approach enabled the correlation of xenon chemical shift values with local geometric features, revealing a clear exponential decay of δ­(^129^Xe) with respect to the mean distance from surrounding atoms. The derived decay constant (∼0.47 Å) and amplitude agree remarkably well with previous studies on simple aliphatic environments, extending their applicability to complex polymeric matrices.

Void space analysis using Zeo++ confirmed the presence of a hybrid pore structure with both isolated and connected cavities. The chemical shift distributions appeared largely independent of Xe loading (1, 3, 10 atoms), consistent with RDF results and previous experimental observations, suggesting that each Xe atom probes an individual microenvironment. The introduction of a secondary reference system for the calculation of the chemical shift (Xe@hexane) significantly reduced the discrepancy between calculated and experimental δ values, highlighting the importance of canceling systematic errors in the calculation of chemical shifts in complex environments. It is also worth to mention that, although it might be tempting to directly correlate the chemical shifts distribution of Figure 8 with the void size distribution of Figure 6, the two profiles show a clear qualitative difference. The chemical shift distribution, in fact, after the maximum, decays to zero while the void size distribution after the first maximum does not, indicating that in the simulation box there is a significant number of relatively large pores. The reason for this apparent discrepancy is that the chemical shift is indeed affected by close contact interactions in small pores, but after the size of the pore is large enough to mimic, as far the xenon chemical shift is concerned, the vacuum, then any further increment of the size cannot be detected by the probe atom. Moreover, even in large pores the van der Waals interaction makes more likely for Xe to be relatively close the side walls of the pore, rather than in the middle. This is the reason why a large number of data points are collected at shorter distances from the pore’s wall and only a few at large distances.

Despite the presence of some limitations in the computational protocol (e.g., the use of a GGA functional, and the cluster extraction protocol, based on a fixed cutoff radius) the consistent agreement between structural metrics, MD-derived distributions, and experimental and calculated ^129^Xe shifts, validates the average morphology of the simulated polymer and confirms the sensitivity of ^129^Xe NMR to local confinement. On the other hand, these results also confirm the power of computational NMR as a protocol for structural elucidation also in cases where the structure to be validated or discarded is not a covalent molecule but the average bulk structure of a complex matrix. The protocol developed here can be applied to other microporous materials and provides a powerful framework for designing membranes with tailored void architectures for transport, sensing, and separation applications.
